# A Comprehensive Analysis of the Role of DSN1 in Pan-Cancer Prognosis and Immunotherapy

**DOI:** 10.7150/jca.111585

**Published:** 2025-04-21

**Authors:** Donggang Xu, Yue Zhang, Maoqia Shen, Xiaolong Cao

**Affiliations:** 1 Department of Oncology, Zhujiang Hospital, Southern Medical University, Guangzhou, 510280, Guangdong, China.; 2 Department of Anesthesiology, Zhujiang Hospital, Southern Medical University, Guangzhou, 510280, Guangdong, China.; 3 Translational Medicine Research Center, Zhujiang Hospital, Southern Medical University, Guangzhou, 510280, Guangdong, China.

**Keywords:** DSN1, pan-cancer, biomarker, prognosis, immunotherapy.

## Abstract

**Background:** Dosage Suppressor of NNF1 (DSN1) is a component of the MIS12 kinetochore complex crucial in the cell cycle process. Recent evidence indicates its close association with cancer progression. The study aims to further explore DSN1's role in cancer.

**Methods:** Using public databases, we investigated the expression patterns of DSN1 in mRNA, protein, and single-cell sequencing data across cancer types. Prognostic associations were assessed through survival analysis, and gene mutation frequencies were compared between high and low DSN1 expression groups. Gene set enrichment analysis was conducted to identify relevant biological pathways. We also examined the correlation of DSN1 with DNA methylation, tumor mutation burden (TMB), microsatellite instability (MSI), immune infiltration, and immune-regulatory genes.

**Results:** Our analysis revealed that DSN1 is consistently overexpressed in tumor cells and actively dividing cells compared to normal tissues. The overexpression of DSN1 showed a significant correlation with either poor or favorable prognosis, depending on the cancer type. Notably, cancers such as COAD, LUAD, and UCEC exhibited high mutation and amplification frequencies in the DSN1-high group. Gene set enrichment analysis identified cell cycle-related pathways as the most significantly associated with DSN1 expression. Furthermore, DSN1 expression was positively correlated with DNA methylation, TMB, and MSI in most cancers. DSN1 was also closely associated with tumor-infiltrating immune cells and immune-regulatory genes, as well as immune therapy response and drug sensitivity.

**Conclusion:** Our findings highlight the importance of DSN1 in tumorigenesis, progression, and immune therapy across various cancer types. Further studies are needed to explore its specific applications in individual cancer types.

## Introduction

Despite decades of research and advancements in cancer care, its impact on global health remains profound, underscoring the urgent need for innovative therapeutic strategies. Globally, approximately 20 million individuals were newly diagnosed with cancer in 2022, resulting in 9.7 million deaths to tumor-related causes 1. Therefore, it is imperative to develop new therapeutic approaches, with a focus on immunotherapies that leverage tumor biomarkers, to improve patient outcomes 2.

The Dosage Suppressor of NNF1 (DSN1) is a protein in MIS12 kinetochore complex, essential for kinetochore assembly and cell cycle progression. It ensures the accurate transmission of genetic material by facilitating proper chromosome segregation. Consequently, dysfunction of DSN1 can lead to genomic instability, a favorable condition for tumorigenesis 3,4. While DSN1 is predominantly studied in cancer, emerging evidence suggests its functional relevance in non-cancerous contexts. For instance, its expression is upregulated in high-risk HPV-infected non-cancerous esophageal tissues, implicating a role in early oncogenic alterations 5. Additionally, a germline-specific splice isoform of DSN1 regulates chromosome segregation fidelity during oocyte maturation and embryonic development, with dyslinked to fertility defects 6. Importantly, DSN1 expression is closely associated with the development of various cancers, including colorectal cancer 7,8, hepatocellular carcinoma 9, breast cancer 10, gastric cancer 11, low-grade glioma 12, and osteosarcoma 13, by influencing key biological processes such as cell proliferation, apoptosis, migration, and invasion. For instance, depletion of DSN1 leads to G2/M phase arrest and impairs the migration, invasion, and anchorage-independent proliferation of colorectal adenoma cells 7. Elevated DSN1 expression is also linked to a poor prognosis in hepatocellular carcinoma 9. While the role of DSN1 in certain malignancies is partially understood, comprehensive research on its role across pan-cancer remains limited.

This study delves into the pan-cancer landscape of DSN1, examining its expression dynamics, its contribution to cancer biology, and its clinical implications across a spectrum of human cancers. First, we analyzed the expression of DSN1, and stratified patients into DSN1 high and DSN1 low groups, and then compared the genetic variations and pathway difference between these two groups. We assessed the relationship between DSN1 expression and patient prognosis, exploring its potential as a pan-cancer biomarker. Furthermore, we investigated its association with the tumor immune microenvironment and evaluated its function in anti-tumor immune responses. Lastly, we explored the potential uses of DSN1 in cancer treatment by integrating predictions for drug sensitivity and immune therapy response.

## Materials and Methods

### Gene, Protein, and Single-Cell Expression Analysis

**Figure 1:**
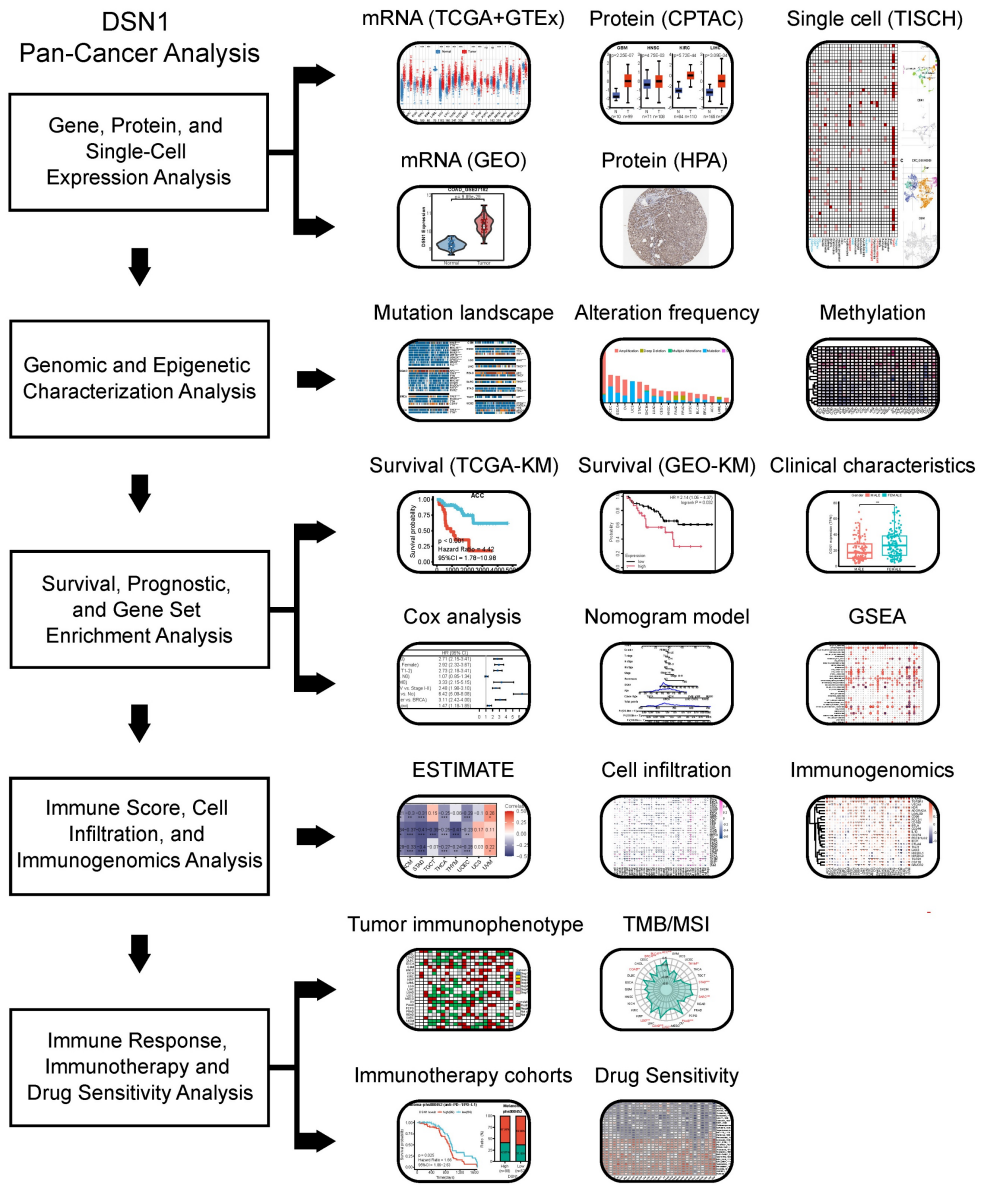
Diagram of main workflow in this study.

DSN1 expression in pan-cancer and normal tissues were obtained from TCGA database, the GTEx database 14, and GEO database. DSN1 expression in normal and pan-cancer tissues were plotted with TIMER2.0 15. The structure and expression levels of DSN1 transcripts were obtained from Ensembl 16 and the UCSC Xena database 17, respectively. Protein levels of DSN1 in various cancer types from the CPTAC database were obtained from the Ualcan database 18. Images of DSN1 stained by immunohistochemistry (IHC) in tumor and normal tissues of different types of cancer were obtained from the Human Protein Atlas (HPA) database 19. The single-cell expression of DSN1 in from various studies were obtained from the TISCH database 20. Data were analyzed using the R software (v4.2.3) for all the statistical analyses. The workflow in this study is demonstrated in Fig. 1.

### Genomic and Epigenetic Characterization Analysis

The pan-cancer gene mutation landscape based on high and low DSN1 expression groups was analyzed using the Comprehensive Analysis on Multi-Omics of Immunotherapy in Pan-Cancer (CAMOIP) platform 21. Alteration frequency statistics for DSN1 were retrieved from the cBioPortal website 22 visualized using the R packages “ggplot2” (v3.5.1) and “ggprism” (v1.5.0). Methylation data from the TCGA database was accessed through the UCSC Xena database 17. The correlation between DSN1 expression and methylation levels was analyzed using custom R scripts with the R package “psych” (v2.4.12) for Spearman correlation.

### Survival and Prognostic Analysis

To validate the clinical prognostic significance of DSN1, expression data and survival data, including overall survival (OS), disease-specific survival (DSS), disease-free interval (DFI), and progression-free interval (PFI), were downloaded from the TCGA database. The optimal cutoff point for DSN1 expression were determined by the R packages “survival” (v3.5-3) based on the OS data and patients with different cancer types were split into DSN1-high and DSN1-low groups accordingly. Cox regression analysis and Kaplan-Meier survival analysis were conducted with the R packages “survival” (v3.5-3) and “survminer” (v0.4.9). Additionally, survival analysis was performed on data from several studies from the GEO database with the KM plot tool 23, generating Kaplan-Meier plots for further evaluate the prognostic importance of DSN1. The relationship between DSN1 expression and clinical information from TCGA, including age, gender, TNM staging, tumor staging (tumor classification based on TNM staging), and recurrence status, was investigated using the t-test. Nomograms were created with the R packages “survival” (v3.5-3), “rms” (v6.8-1), and “regplot” (v1.1), and calibration curves were generated to assess the calibration performance of the nomograms. Furthermore, the receiver operating characteristic (ROC) curves analyses were performed with the R package “survivalROC” (v1.0.3.1).

### Gene Set Enrichment Analysis (GSEA)

Patients from different TCGA cohorts were divided to DSN1 high and low groups based on the top and bottom 30% of DSN1 expression levels. The differential gene expression analysis between the two groups were then performed with the Wilcoxon test. The MSigDB database was used to retrieve the hallmark gene sets (h.all.v2023.2.Hs.symbols.gmt). The R package “clusterProfiler” 24 (v4.14.4) was used to run GSEA on the differential gene expression results.

### Immune Score, Immune Cell Infiltration and Immunogenomics Analysis

The R package “estimate” 25 (v1.0.13) were used to compute immune score, stromal score, and ESTIMATEScore (a combined score of immune and stromal components) for various tumor samples. Immune infiltration abundance data for different cell types across 32 cancer types was downloaded from the TIMER2.0 database 15. Spearman correlation analysis was conducted to assess the relationship between DSN1 expression levels and immune/stromal scores, as well as the correlation between DSN1 expression and the immune infiltration abundance of diverse cell types. Additionally, the co-expression of immune regulatory genes with DSN1 was analyzed.

### Anti-cancer Immune Response, Immune Therapy and Drug Sensitivity Analysis

The anti-cancer immune activity ratings across the cancer immune cycle were compared and analyzed between high and low DSN1 expression groups across 33 cancer types using the Tracking Tumor Immunophenotype (TIP) database 26. Spearman correlation analysis was performed to evaluate the association between DSN1 expression levels and tumor mutational burden (TMB) or microsatellite instability (MSI). Radar plots were produced using the R package “fmsb” (v0.7.6). DSN1 expression data and associated clinical information for SKCM and BLCA patients undergoing anti-PD-1/PD-L1 treatment were obtained from the dbGaP (phs000452) 27 and IMvigor210 datasets 28. Survival analysis was conducted using the R packages “survival” (v3.5-3) and “survminer” (v0.4.9). The relationship between DSN1 expression and tumor cell susceptibility to anti-cancer drugs was analyzed using the R package “oncoPredict” 29 (v1.2) and data from the Genomics of Drug susceptibility in Cancer (GDSC) database 30.

## Results

### Elevated DSN1 Expression in Cancer Tissues Compared to Normal Tissues

**Figure 2:**
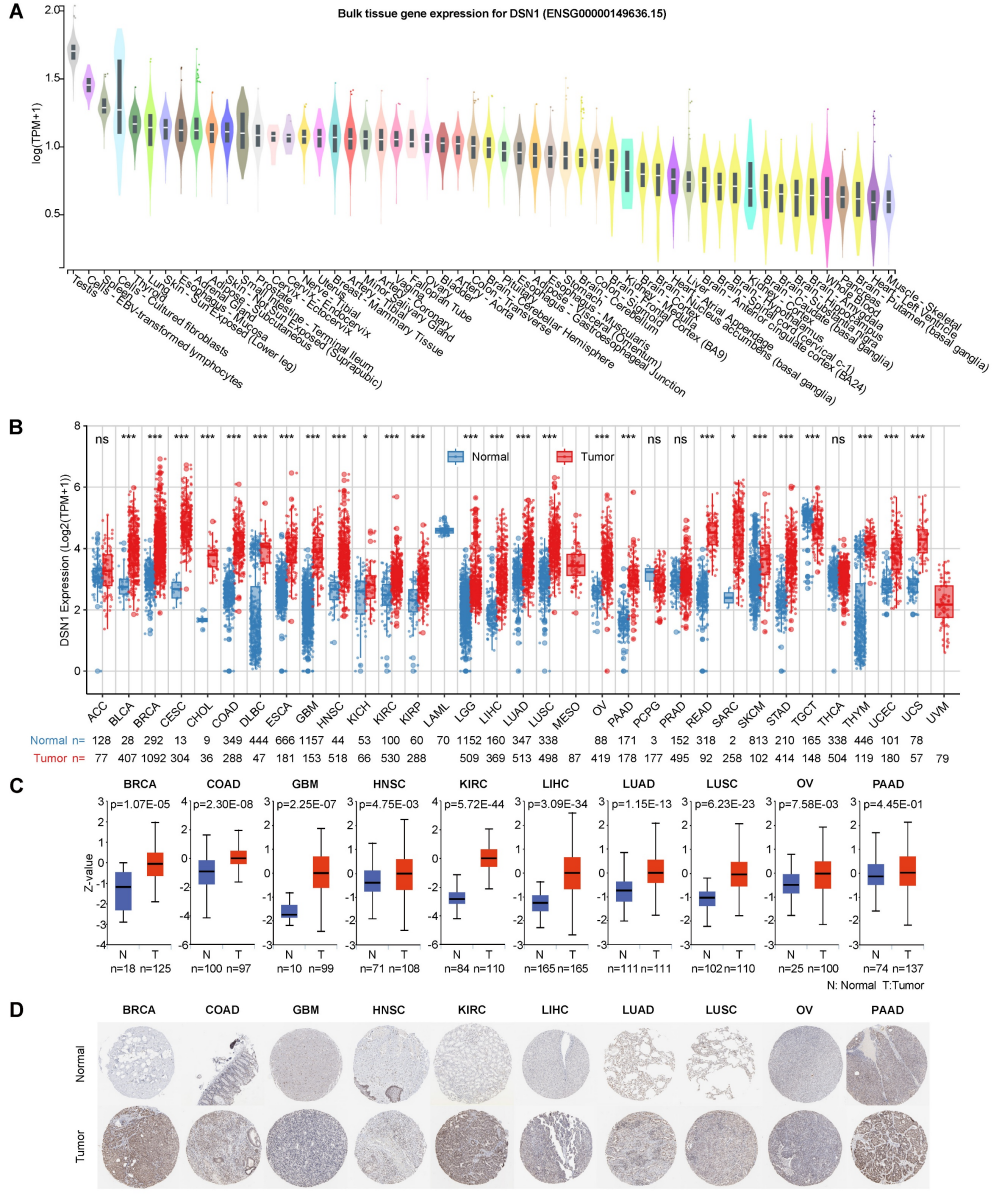
DSN1 expression levels in normal and tumor tissues of humans. (A) Violin plot showing DSN1 expression levels in various human normal tissues. (B) Box plot showing DSN1 mRNA expression levels in normal and tumor tissues, derived from TCGA and GTEx datasets, with statistical significance assessed by Wilcoxon test. (C) Box plot illustrating DSN1 protein levels in normal and tumor tissues, with blue and red representing normal and tumor tissues, respectively. (D) Representative immunohistochemistry staining images of DSN1 in 10 types of normal and tumor tissues. The symbols ns, *, **, and *** represent not significant, p < 0.05, p < 0.01, and p < 0.001, respectively.

We first examined DSN1 mRNA expression levels in normal tissues using data from the GTEx database 14. Relative higher expression levels of DSN1 were observed in tissues with cell cycle activity such as the testes, spleen, thyroid, lungs, and skin, while DSN1 expression was lower in non-proliferative organs such as the heart and brain (Fig. 2A). Single-cell RNA sequencing revealed that DSN1 was highly expressed in various epithelial cells across normal tissues (Fig. S1A). With data from the TCGA and GTEx databases, we found that DSN1 expression was significantly elevated in most cancer samples compared to normal samples (Fig. 2B and S1B). Additionally, data from twelve independent studies in the GEO database 31,32 consistently confirmed that DSN1 was upregulated in most cancers types (Fig. S1C). The analysis of 10 DSN1 transcripts, including 7 protein-coding, 3 non-coding ones showed that, almost all protein-coding transcripts were upregulated in most tumor tissues, while non-coding ones did not exhibit a clear pattern of upregulation or downregulation (Fig. S2).

Next, we utilized the CPTAC protein expression data from the UALCAN database 18 to compare DSN1 protein levels in normal and tumor tissues. The results showed that DSN1 protein expression was considerably elevated in 9 cancer types, including BRCA, COAD, GBM, HNSC, KIRC, LIHC, LUAD, LUSC, and OV (Fig. 2C). Furthermore, IHC images in the HPA database 19 corroborated these findings, showing higher DSN1 protein signals in the cancer tissues compared to the normal counterparts (Fig. 2D).

**Figure 3:**
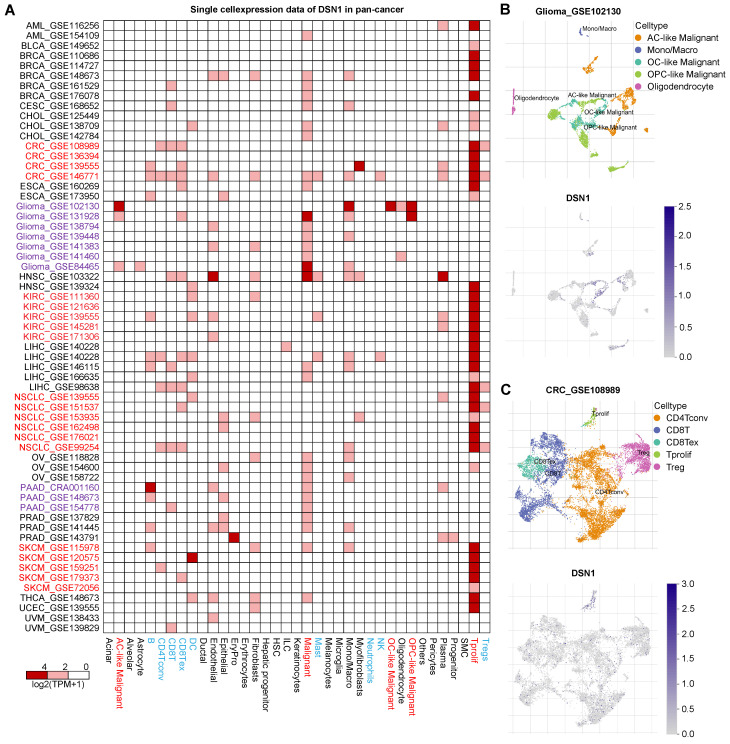
Single-cell expression analysis of DSN1 in tumor tissues. (A) Cluster heatmap showing mRNA levels of DSN1 across 37 cell types in tumor tissues. (B-C) Umap plots illustrating the clustering of different cell types (upper panel) and DSN1 expression levels (lower panel) in Glioma (B) and CRC (C) tissues.

To investigate DSN1 expression at single-cell resolution, we analyzed a large dataset of single-cell RNA-sequencing profiles from the TISCH database, encompassing 61 studies across 19 different cancer type 20. Our findings revealed that DSN1 exhibited elevated expression levels in both malignant and immune cells, with particularly notable expression in proliferating T cells (Tprolif) in many cancer types (Fig. 3A). For example, in most glioma and Pancreatic adenocarcinoma (PAAD) datasets, DSN1 was predominantly expressed in malignant cells as well as other cell types (Fig. 3B). In most CRC, KIRC, NSCLC, and SKCM datasets, DSN1 was higher expressed in Tprolif cells (Fig. 3C). We should notice that DSN1 was not found to be highly expressed in malignant cells in some studies because these studies focused on specific cells, and did not include a comprehensive analysis of all cell types. Collectively, these data suggest that DSN1 may be actively involved in the cellular processes that underpin tumor development.

### Pan-Cancer Genomic and Epigenomic Differences Based on DSN1 Expression

**Figure 4:**
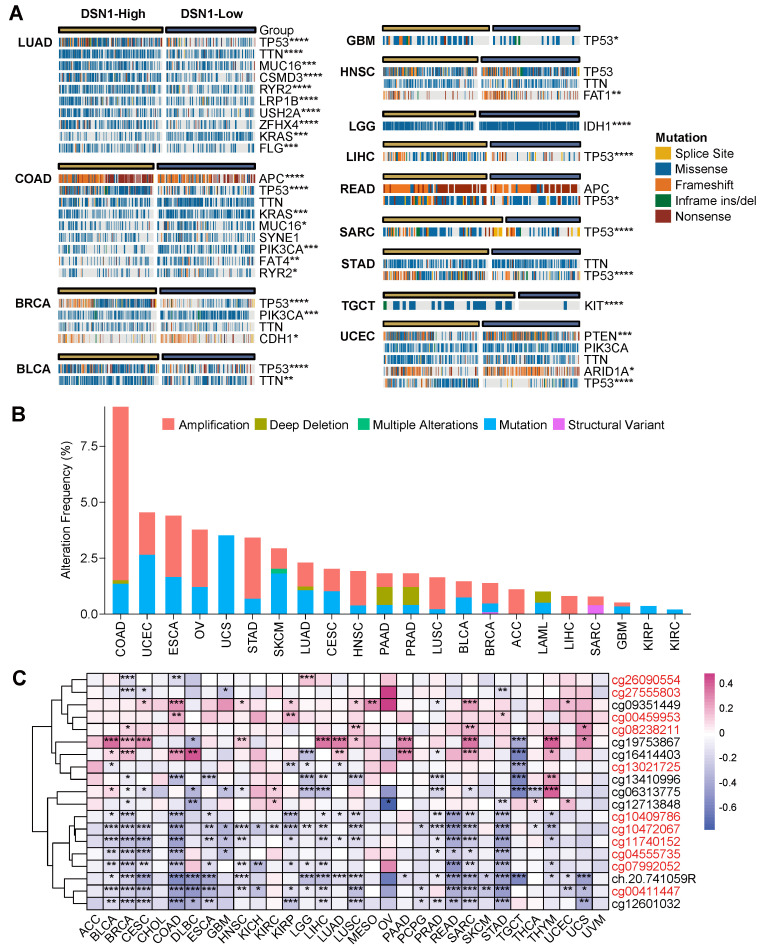
Genomic epigenetic variation profiling based on DSN1 expression. (A) A panorama of the top 10 frequently mutated genes with significant differences between high-expression and low-expression groups based on the median DSN1 expression across pan-cancer. Fisher's exact test and BH-corrected p-values were applied. (B) Alteration frequency of DSN1 across various cancer types. (C) DNA methylation analysis of DSN1 across 32 cancer types, highlighting probes targeting the promoter region (red ID probes). The symbols *, **, ***, and **** represent p < 0.05, p < 0.01, p < 0.001, and p < 0.0001, respectively.

To determine the pan-cancer genomic and epigenetic characteristics of DSN1, we examined the variations in the pan-cancer mutation landscape between the DSN1-high and DSN1-low expression groups using the CAMOIP platform 21. Our findings revealed significant differences in mutation frequencies for several key genes across various tumors. Notably, the frequency of TP53 mutations varied significantly between the DSN1-high and DSN1-low expression groups in many cancer types, including LUAD, COAD, BRCA, BLCA, and others (Fig. 4A).

Furthermore, we used the cBioPortal database to investigate the mutation types and alteration frequency of DSN1 across pan-cancer. The results demonstrated significant differences in alteration frequencies of DSN1 among different cancer types, with COAD exhibiting the highest mutation frequency (approximately 9.26%), followed by UCEC and ESCA. Amplification was the most common mutation type for DSN1, with high amplification frequencies observed in COAD, ESCA, OV, and STAD. Additionally, deep deletion was observed in COAD, LUAD, PAAD, PRAD, and LAML, while multiple alterations were only found in SKCM (Fig. 4B).

Methylation sites of DSN1, analyzed using TCGA data, were primarily located near the Transcription Start Site (TSS) and in downstream regions (Fig. S2D). Correlation analysis across various cancer types indicated that methylation at most TSS-proximal sites negatively correlated with DSN1 expression, suggesting a regulatory role in gene suppression. Notably, methylation at cg19753867 revealed a markedly positive correlation with DSN1 expression in nearly 40% of cancer types, including BLCA, BRCA, and others (Fig. 4C).

### DSN1 as a Prognostic Biomarker Across Multiple Cancer Types

**Figure 5:**
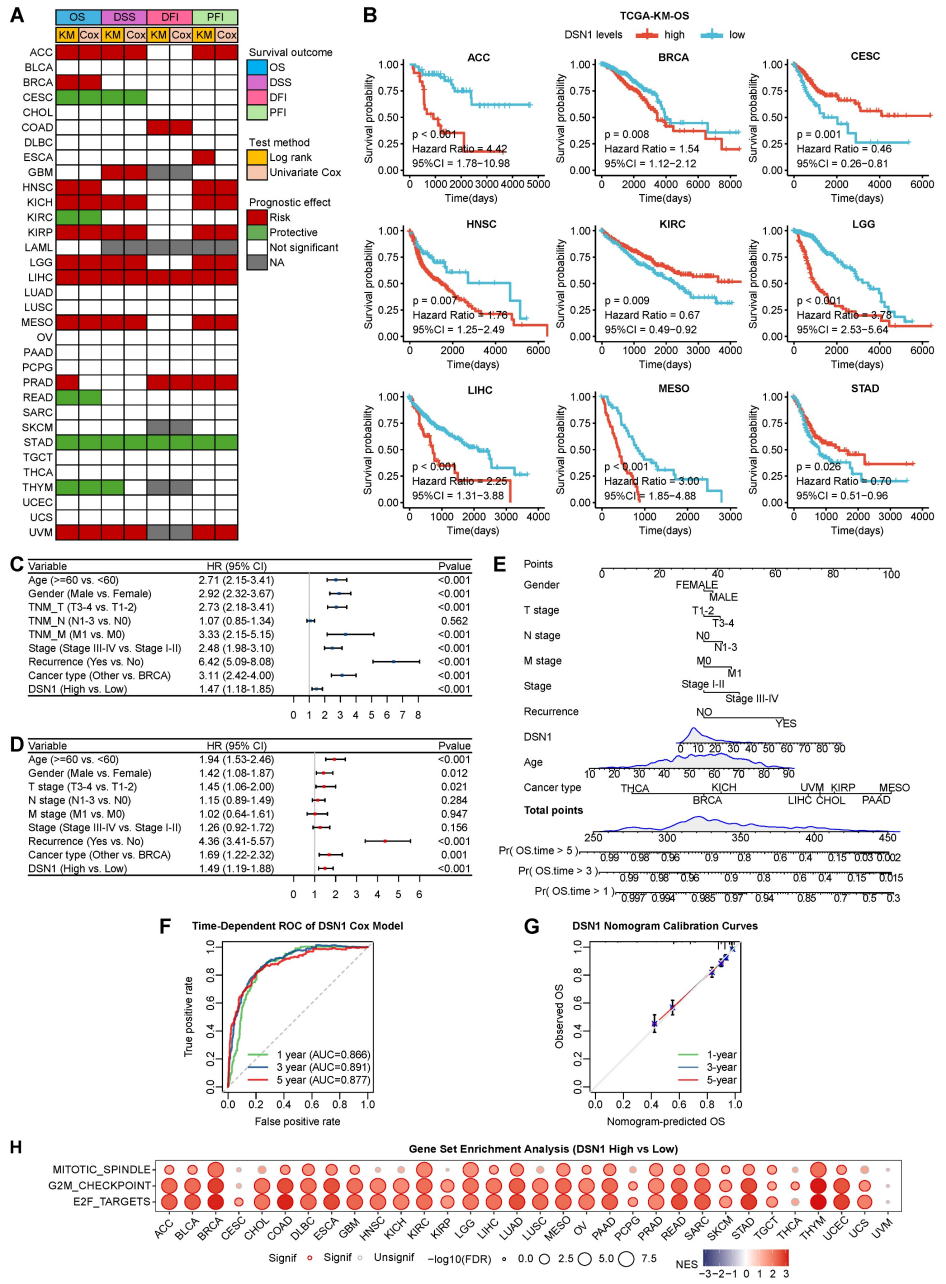
Prognostic and GSEA analysis based on DSN1 expression. (A) Heatmap showing the correlation between DSN1 expression levels and for survival outcomes (OS, DSS, DFI, PFI), derived from the TCGA database. Survival analysis was performed using Kaplan-Meier (KM) test and univariate Cox regression. Red, green, white, and gray boxes indicate risk factors, protective factors, non-significant analysis, and unavailable data, respectively. (B) Survival curves comparing the prognosis of DSN1-high and DSN1-low expression groups across 9 cancers in the TCGA database. (C) Univariate Cox regression analysis of DSN1. (D) Multivariate Cox regression analysis of DSN1. (E) Nomogram for predicting 1-year, 3-year, and 5-year overall survival rates for pan-cancer patients. (F) Time-dependent ROC curves of the DSN1 Cox regression model predicting 1-year, 3-year, and 5-year overall survival. (G) Calibration plots for the nomogram predicting 1-year, 3-year, and 5-year overall survival. (H) Bubble plot of GSEA results between high and low DSN1 expression in tumors using hallmark gene sets. Circle size represents p-value magnitude, and color gradient (red to blue) indicates normalized enrichment scores (NES).

To evaluate the clinical prognostic significance of DSN1 across various cancer types, we conducted a comprehensive analysis of its association with overall survival (OS), disease-specific survival (DSS), disease-free interval (DFI), and progression-free interval (PFI) using data from 33 cancer types in the TCGA database. The results indicated that DSN1 overexpression was related to poorer OS and DSS in multiple cancer types, including ACC, BRCA, HNSC, KICH, KIRP, LGG, LIHC, MESO, PRAD, and UVM (Fig. 5A, 5B, and S3A). Conversely, high DSN1 expression correlated with better OS and DSS in patients with CESC, KIRC, READ, STAD, and THYM. Additionally, elevated DSN1 level were identified as a risk factor for DFI and PFI in various cancer types (Fig. 5A and S3A). Kaplan-Meier survival analysis using multiple GEO datasets confirmed that high DSN1 expression was associated with poor OS in patients with BRCA, COAD, LAML, LUAD, OV, and PAAD, while it served as a protective factor for OS in patients with LUSC and STAD (Fig. S3B). Moreover, TCGA phenotype data analysis indicated that DSN1 expression was associated with several clinical features in cancer patients (Fig. S4). Higher DSN1 levels were found in older patients with BLCA, LGG, and UVM. Significant gender-based differences in DSN1 expression are observed in DLBC, HNSC, SARC, and SKCM. DSN1 expression is associated with proliferation and invasion (T stage) in several cancers, including HNSC, LIHC, LUAD, PRAD, TGCT, and THCA. Additionally, it is linked to regional lymph node metastasis (N stage) in ACC, HNSC, and PRAD. Interestingly, in CESC, LIHC, and STAD, higher DSN1 levels are negatively correlated with distant metastasis (M stage). DSN1 expression was associated with tumor staging in ACC, KIRP, LIHC, and SKCM, and high DSN1 levels were linked to increased recurrence risk in ACC, BLCA, LGG, LIHC, PRAD, SARC, and UVM.

To assess the predictive effect of DSN1 across pan-cancer types, we employed univariate and multivariate Cox regression analyses, which revealed that DSN1 expression, along with age, gender, TNM T stage, TNM M stage, tumor stage, recurrence, and cancer type, independently predicted patient survival (Fig. 5B and 5C). Based on multivariate Cox regression and clinical factors, a nomogram was conducted to predict the 1-, 3-, and 5-year overall survival for pan-cancer patients (Fig. 5E). The predictive ability of the model was validated using receiver operating characteristic (ROC) curves (Fig. 5F), and the nomogram's effectiveness was confirmed by calibration curves (Fig. 5G).

### DSN1 Overexpression Correlates with Cell Proliferation Pathways

We performed a pan-cancer Gene Set Enrichment Analysis (GSEA) to compare differentially expressed genes (DGEs) between high and low DSN1 expression groups across various cancer types to investigate the influence of DSN1 in cancer development. High DSN1 expression in most cancer types was notably enriched in cell proliferation-related signaling pathways, such as MYC (which drives transcriptional activation of oncogenic targets) 33, mTORC1 (central to nutrient sensing and mitotic progression) 34, Mitotic spindle (regulating spindle formation), G2M (a critical phase of mitosis regulated by DNA damage response proteins) 35, and E2F (transcription factors controlling S-phase entry and cell cycle progression) 36 pathways (Fig. 5H and S5). High DSN1 expression (as a centromere component) is associated with the activation of cell proliferation pathways and cell cycle progression, potentially promoting genomic instability that triggers DNA damage checkpoints or apoptosis, thereby contributing to tumor growth and progression 37. This association is consistent with the function of DSN1 as a centromere protein, which plays a critical role in cell division and chromosome segregation.

### DSN1 Associates with Reduced Immune and Stromal Activity

**Figure 6:**
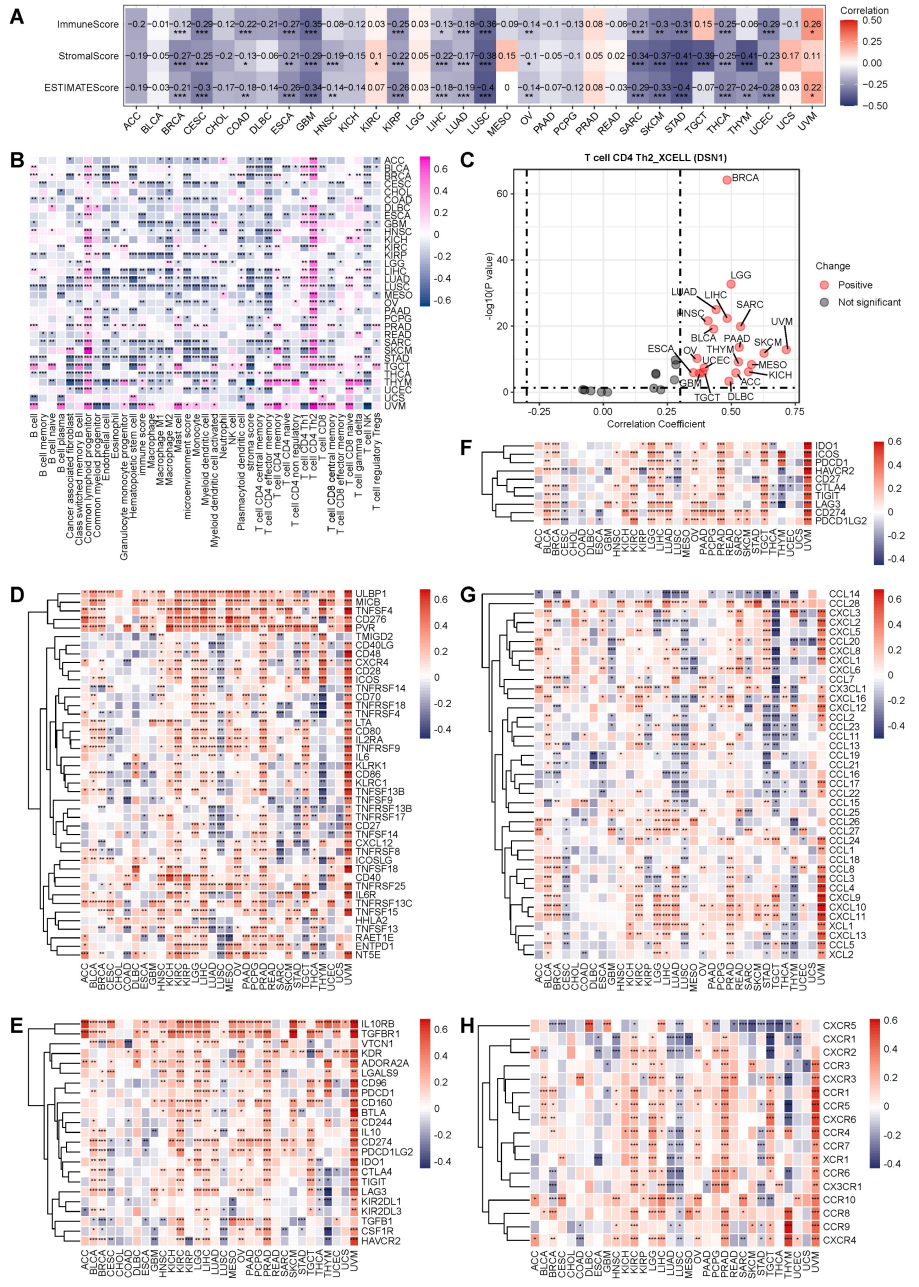
Pan-cancer immune correlation analysis of DSN1. (A) Heatmap showing the correlation between DSN1 expression and immune score, stromal score, and ESTIMATEScore calculated by the R package “estimate” (v4.14.4). (B) Heatmap displaying the correlation between DSN1 expression and immune cell infiltration levels, based on the XCELL algorithm. (C) Scatter plot showing the Spearman correlation analysis between DSN1 expression and Th2 cell infiltration in pan-cancer using the XCELL algorithm. (D-H) Heatmaps illustrating the Spearman correlation results between DSN1 expression and genes related to immune stimulatory factors (D), immune suppressive factors (E), immune checkpoints (F), chemokines (G), and chemokine receptors (H) across pan-cancer. The symbols *, **, and *** represent p < 0.05, p < 0.01, and p < 0.001, respectively.

To investigate the immune-related role of DSN1 across various cancers, we initially assessed the relationship between DSN1 expression and immune and stromal scores across 32 cancer types. The results demonstrated a significant negative correlation (p<0.01) between DSN1 expression and these scores in several cancers, including BRCA, CESC, COAD, ESCA, GBM, HNSC, KIRP, LIHC, LUAD, LUSC, OV, SARC, STAD, THCA, THYM, and UCEC (Fig. 6A). Furthermore, we explored the correlation between DSN1 levels and immune cell infiltration across multiple cancer types. The analysis revealed a negative correlation between DSN1 expression and the infiltration of various immune cells, as determined by the XCELL algorithm, except for CD4+ T helper 2 (Th2) cells and common lymphoid progenitors, which showed a positive association (Fig. 6B and 6C).

Additionally, a co-expression analysis of DSN1 and immune-related genes indicated that DSN1 expression was positively correlated with several immune-stimulatory genes, including ULBP1, MICB, TNSF4, CD276, PVR, and TNFRSF13C, among 43 immune-stimulatory genes (Fig. 6D). Moreover, DSN1 was positively correlated with 23 immune-suppressive genes, including IL10RB, TGFBR1, KDR, ADORA2A, and CD274 (Fig. 6E). DSN1 expression also showed significant associations with immune checkpoint genes, chemokines, and chemokine receptors (Fig. 6F-H). Notably, DSN1 expression showed a negative correlation with many immune-related genes in LUSC, which is different from many other cancer types. These findings suggest a complex and multifaceted co-expression pattern between DSN1 and immune-related genes.

### DSN1 as a Prognostic Biomarker in Tumor Immunotherapy and Predictor of Drug Sensitivity

**Figure 7:**
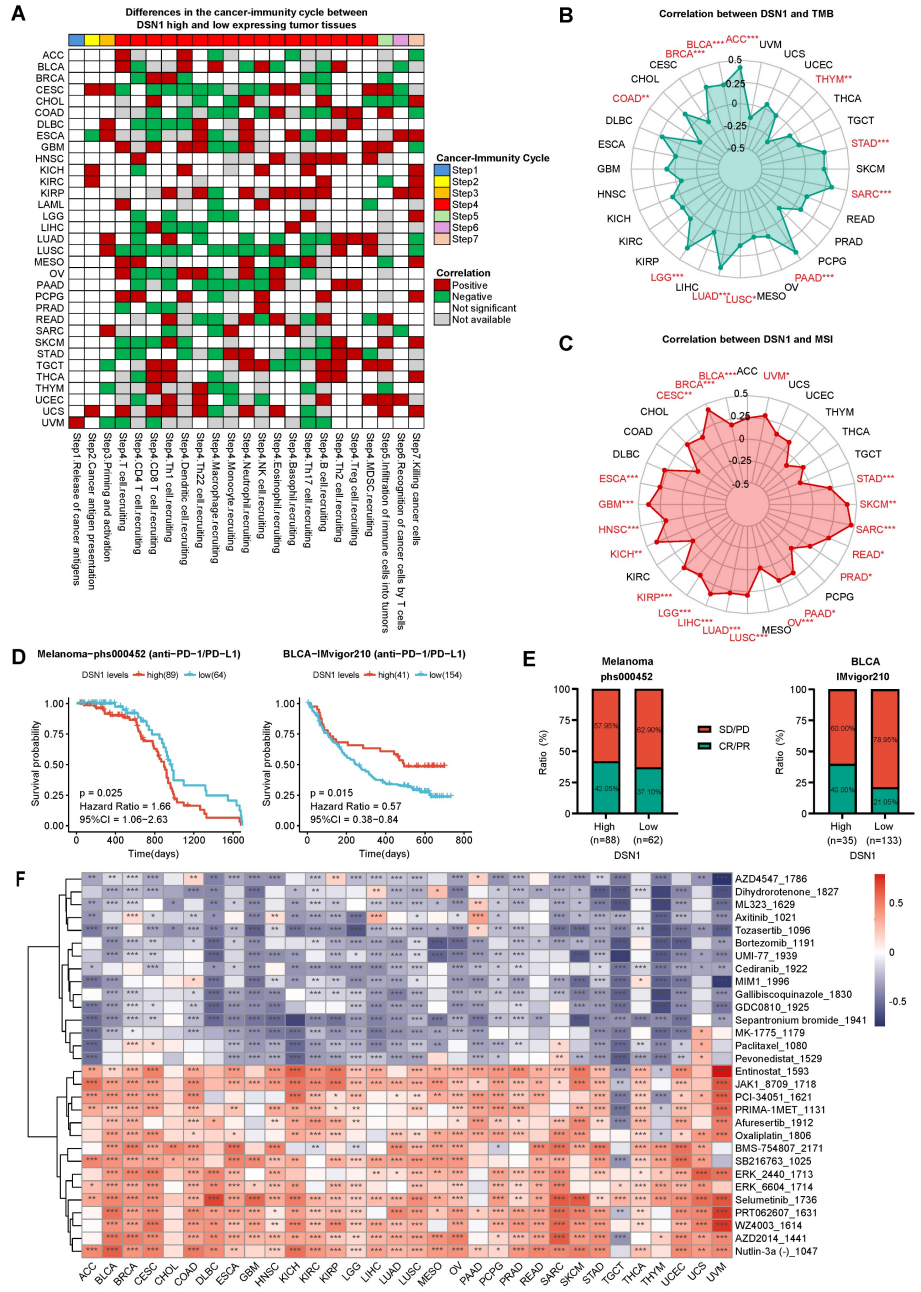
Immune therapy and drug sensitivity analysis of DSN1. (A) Heatmap clustering showing the differences in cancer immune cycle stages between DSN- high and DSN1-low expression groups. Patients were categorized into DSN1-high and DSN1-low expression groups based on their DSN1 expression levels, with the top 30% of patients classified as DSN1-high and the bottom 30% classified as DSN1-low. Those with p < 0.05 were considered significant. Red boxes indicate positive correlations, green boxes indicate negative correlations, and white boxes represent non-significant correlations. (B-C) Radar plots showing the correlation between DSN1 expression and TMB (B) and MSI (C). (D) Predictive value of DSN1 expression on overall survival (OS) in Melanoma (left) and BLCA (right) patients receiving anti-PD-1/PD-L1 immunotherapy. (E) Immune response rates in Melanoma (left) and BLCA (right) patients. PD, progressive disease; SD, stable disease; CR, complete response; PR, partial response. (F) Heatmap of the correlation between DSN1 expression and sensitivity scores for various anticancer drugs. Red boxes indicate positive correlations (high DSN1 expression associated with increased drug resistance), while blue boxes represent negative correlations (high DSN1 expression associated with increased drug sensitivity), with lower drug sensitivity scores reflecting higher drug sensitivity. The symbols *, **, and *** represent p < 0.05, p < 0.01, and p < 0.001, respectively.

To elucidate the role of DSN1 expression in pan-cancer immunotherapy, we utilized the TIP database to obtain immune activity ratings for the cancer immune cycle. Our findings indicated that high DSN1 levels were positively correlated with neutrophil recruitment (step 4), Th2 cell recruitment (step 4), and cancer cell killing (step 7). Conversely, in most cancer types, elevated DSN1 expression was negatively correlated with CD4⁺ T cell recruitment (step 4), macrophage recruitment (step 4), and Th17 cell recruitment (step 4) (Fig. 7A). The observed reduction in CD4⁺ T cells, macrophages, and Th17 cells 38 may impair anti-tumor immune surveillance, as these cells are crucial for antigen presentation, modulation of the tumor microenvironment, and inflammatory responses. Additionally, in patients with CESC, COAD, and LUSC, high DSN1 levels were negatively associated with immune activity scores at several stages of the immune cycle.

To further explore the significance of DSN1 in tumor microenvironment (TME) immunotherapy, we analyzed the correlation between DSN1 expression and tumor mutational burden (TMB) as well as microsatellite instability (MSI). The results revealed a positive correlation between DSN1 expression and TMB scores in ACC, BLCA, BRCA, LGG, LUAD, LUSC, PAAD, SARC, and STAD, whereas a negative correlation was observed in COAD and THYM (Fig. 7B). Additionally, DSN1 expression exhibited a positive correlation with MSI scores in many cancer types including ACC, BLCA, BRCA, CESC, ESCA, and others (Fig. 7C).

Furthermore, we explored the potential predictive role of DSN1 in anti-PD-1/PD-L1 therapy by analyzing immune therapy cohorts from the dbGaP (phs000452) and IMvigor210 datasets. The analysis revealed that patients with high DSN1 expression had a longer overall survival (OS) in melanoma, while a shorter OS was observed in BLCA (Fig. 7D). Additionally, patients with high DSN1 expression in melanoma and BLCA exhibited higher response rates to immunotherapy (Fig. 7E). These results indicate that DSN1 may be a useful biomarker for predicting the effectiveness of immunotherapy.

To evaluate the role of DSN1 in drug sensitivity screening, we used the GDSC v2 database to calculated sensitivity scores for anticancer drugs and conducted a correlation analysis. The findings revealed a strong correlation between DSN1 expression and various anticancer drugs across multiple cancer types (Fig. 7F). In nearly all cancer types, high DSN1 expression was significantly negatively correlated with Tozasertib and Sepantronium (indicating high drug sensitivity) and significantly positively correlated with Selumetinib (indicating high drug resistance). Interestingly, Tozasertib, an Aurora kinase and BCR-ABL inhibitor evaluated in early-phase clinical trials for refractory hematologic malignancies, demonstrated hematologic responses in Phase I/II studies 39. Similarly, Sepantronium, a survivin inhibitor studied in Phase I trials for advanced solid tumors and lymphomas, achieved preliminary antitumor activity with defined maximum tolerated doses and manageable toxicity 40. These findings align with our analysis and suggest that DSN1 has potential clinical applications in selecting anticancer drugs.

## Discussion

Cancer's high global mortality rate drives the imperative for developing new and effective therapeutic strategies and the identification of reliable biomarkers for early detection, prognosis, and treatment response prediction 41,42. While the crucial role of DSN1 in maintaining genomic stability through proper chromosome segregation is established 37,43, its pan-cancer implications remain under investigation. Previous studies have linked DSN1 overexpression to poor prognosis in specific cancers, such as colorectal cancer 7, liver cancer 9, breast cancer 10 and lower-grade glioma 12. This study provides the first comprehensive pan-cancer analysis of DSN1, revealing its diverse roles in tumorigenesis and progression, and establishing its potential as a promising biomarker for immunotherapy and drug sensitivity prediction (Table 1, Fig. S6).

Our findings, in conjunction with previous research, demonstrate that DSN1 is frequently overexpressed in a wide array of cancers. This overexpression is evident at both the mRNA and protein levels (Table 1), as shown by the analysis of TCGA, GTEx, and CPTAC databases. The elevated expression of DSN1 in both cancerous tissues and normal tissues with high cell turnover, further supports its role in actively dividing cells and its potential contribution to tumorigenesis. The consistent upregulation across diverse cancer types, highlights its potential as a common driver of oncogenesis.

The observed upregulation of DSN1 in cancer is likely driven by a combination of genomic and epigenomic alterations. Our analysis reveals that gene amplification is a significant contributor, particularly in cancers like COAD, ESCA, OV, and STAD, where high frequencies of DSN1 amplification were observed. Furthermore, the differential TP53 mutation frequencies between DSN1-high and DSN1-low groups in several cancers, including LUAD, COAD, BRCA, BLCA, GBM, LIHC, READ, SARC, and UCEC, intimate a complex interplay between DSN1 and this crucial tumor suppressor. DSN1 overexpression caused genomic instability may create a cellular environment that selects for TP53 mutations, thereby compromising its ability to safeguard genomic integrity. The observed negative correlation between methylation at TSS-proximal sites and DSN1 expression suggests that DNA methylation likely plays a role in suppressing DSN1 levels, adding another layer of regulation to its expression.

The prognostic significance of DSN1 exhibits marked variability across different cancer types. While elevated DSN1 expression is associated with poor prognosis in numerous cancers, including ACC, BRCA, HNSC, KICH, KIRP, LGG, LIHC, MESO, PRAD, and UVM, it paradoxically correlates with a favorable prognosis in others, such as CESC, KIRC, READ, STAD, and THYM. This dichotomy likely reflects the diverse tumor microenvironments and the specific oncogenic pathways that predominate in different cancer types. In cancers where DSN1 overexpression drives aggressive proliferation and genomic instability (e.g., BRCA LIHC), its association with poor outcomes is consistent with its role in promoting uncontrolled cell division. Conversely, in cancers like KIRC and STAD, where other oncogenic pathways might be more dominant or where a higher degree of differentiation is maintained, high DSN1 expression could reflect a less aggressive subtype or distinct underlying biology where excessive DSN1 triggers immunogenicity (via elevated TMB/MSI) and mitotic catastrophe (through unresolved chromosome missegregation), tipping the balance toward tumor suppression 44. These findings underscore the importance of considering the specific cellular and molecular context when evaluating DSN1's prognostic value.

Across multiple cancer types, high DSN1 expression was significantly associated with the activation of key cell cycle-related pathways, suggesting that DSN1 may promote tumor cell proliferation by influencing these pathways. This aligns with previous studies that DSN1 directly promotes colorectal cancer progression by regulating the G2/M phase of the cell cycle 7. Previous study in gastric cancer demonstrated that silencing of the transcription factor estrogen-related receptor alpha (ESRRA) downregulates DSN1, leading to inhibition of the CDC25C-CDK1-Cyclin B1 pathway and subsequent G2/M arrest 45. While ESRRA is recognized for its role in various cancers, including breast cancer 46, glioma 47, and gallbladder cancer 48, its regulation of DSN1 provides a mechanistic link between this transcription factor and the cell cycle machinery. This highlights DSN1 as a potential key downstream target of ESRRA, mediating its oncogenic effects through modulation of cell cycle pathways.

The relationship between DSN1 and the tumor immune microenvironment exhibited striking variations across different cancer types. While high DSN1 expression in many cancers correlates negatively with immune and stromal scores, the underlying mechanisms appear to be cancer-type specific. In most cases, high DSN1 is associated with reduced infiltration of most immune cell types, potentially facilitating immune evasion by rapidly proliferating tumor cells. However, the notable positive correlation with Th2 cell infiltration across various cancers suggests another potential mechanism by which DSN1 may promote tumor progression. Th2 cells, known for their immunosuppressive functions and ability to dampen cytotoxic T cell responses, could create a feedback loop favoring tumor growth in DSN1-high tumors 49. The distinct immune landscape of LUSC, where DSN1 is negatively correlated with many immune-related genes, further emphasizes this cancer-type specificity. The heterogeneity in DSN1's interaction with the immune landscape underscores the necessity for a context-dependent understanding of its immunomodulatory role in different cancers.

Immune checkpoint inhibitor (ICI)-based immunotherapy has emerged as a highly promising strategy in cancer treatment 50, and DSN1 showed a potential as a biomarker for immunotherapy and drug sensitivity. The positive correlation between DSN1 expression and TMB, as well as MSI, in a substantial number of cancers suggests that DSN1-high tumors might be more immunogenic, potentially due to increased neoantigen presentation resulting from genomic instability. This could, in part, explain the higher response rates to immunotherapy observed in DSN1-high patients with melanoma and BLCA (Fig. 7E). However, the contrasting prognostic implications of high DSN1 expression in these two cancers during immunotherapy—favorable in BLCA and unfavorable in SKCM—highlight the critical influence of the specific tumor immune microenvironment. In BLCA, DSN1 correlates positively with CD4+ Th1 cells (Fig. 6B), PDCD1, LAG3 (Fig. 6F), CXCR5 (Fig. 6H), and enhanced T-cell recruitment (Fig. 7A), indicative of a pro-inflammatory and immunoresponsive microenvironment. Conversely, in SKCM, DSN1 is associated with reduced Th1 infiltration (Fig. 6B), downregulated PDCD1 and LAG3 (Fig. 6F), suppressed CXCR5 (Fig. 6H), and impaired T-cell recruitment (Fig. 7A), collectively fostering an immunosuppressive milieu resistant to checkpoint inhibition. Furthermore, the association between DSN1 expression and drug sensitivity, particularly the increased sensitivity to Tozasertib and Sepantronium in DSN1-high tumors, provides a compelling rationale for exploring DSN1 as a predictive biomarker for selecting personalized therapeutic strategies. The fact that these drugs target pathways involved in apoptosis, cell proliferation, and protein degradation aligns with DSN1's functions in cell cycle regulation and suggests a mechanistic link between DSN1 expression and drug response. In particular, Tozasertib, an Aurora kinase inhibitor, is hypothesized to interfere with the Aurora B phosphorylation of DNS1, thereby inhibiting the CENP-C:MIS12-C interaction and ultimately blocking mitosis and tumor proliferation 51,52.

Our study provides a comprehensive pan-cancer analysis of DSN1, revealing its multifaceted roles in tumorigenesis and its context-dependent impact on prognosis and the tumor immune microenvironment (Table 1, Fig. S6). While these findings are promising, they require validation through *in vitro* and *in vivo* experiments with more clinical data, as well as further investigation with larger clinical datasets to confirm the potential differential roles of DSN1 across various cancer types.

## Conclusion

DSN1 emerges as a critical player in cancer with multifaceted clinical implications: (a) as a prognostic biomarker, its dual prognostic significance (poor outcome in aggressive cancers like BRCA/LIHC versus favorable prognosis in KIRC/STAD) reflects tumor microenvironment heterogeneity; (b) a as an immunotherapy response indicator, where its association with elevated TMB/MSI and differential immune microenvironment features (e.g., Th1/Th2 balance, PDCD1/LAG3 expression) predicts response variability in melanoma and bladder cancer; and (c) as a therapeutic target, with DSN1-high tumors showing heightened sensitivity to mitotic kinases inhibitors (Tozasertib) and survivin antagonists (Sepantronium). These context-specific roles, combined with its impacts on genomic instability, immune modulation, and drug sensitivity, establish DSN1 as a versatile candidate for precision oncology strategies to improve diagnostic and therapeutic outcomes.

## Supplementary Material

Supplementary figures.

## Figures and Tables

**Table 1:** Summary of the multidimensional biological roles and associations of DSN1 across various cancers. Column “mRNA”, mRNA expression comparison between normal and tumor tissues. “+”/“-” means significant upregulation/downregulation based on TCGA/GEO studies while “++” means upregulation in both. Column “Pr.”, DSN1 protein levels in tumor vs. normal tissues. “+” means significant upregulation based on CPTAC studies. Column “SC”, single cell analysis. “T” means higher expressed in proliferating T cells while “M” means in malignant cells. Column “TP53 Mut.”, “+” means TP53 mutation were significantly different in DSN1 high and DSN1 low groups. Column “KM OS”, Kaplan-Meier overall survival analysis of DSN1 high and low expression groups. “+”/“-” indicates DSN1 as a risk/protective factor based on TCGA/GEO studies, while “++”/“--” denotes DSN1 as a risk/protective factor in both. Column “Met”, methylation levels. “-” indicates a negative correlation between DSN1 expression and methylation levels. Column “MSI”, Microsatellite Instability. “+” denotes a positive correlation between DSN1 expression and MSI (p < 0.05). Column “Th2”, “+” indicates a positive correlation between DSN1 and Th2 immune infiltration score based on the XCELL algorithm (p < 0.05). Column “ES”, ESTIMATEScore. “-” represents a negative correlation with DSN1 (p < 0.05). Column “Mac.”, macrophage recruitment. “+”/“-” means a positive/negative relationship between DSN1 and macrophage recruitment (p < 0.05). Columns “IF”, “ISTF”, “ISUF”, “ICP”, “CK”, and “CKR” represent immune cell infiltration, immune stimulatory factors, immune suppressive factors, immune checkpoints, chemokines, and chemokine receptors, respectively. Values indicate the difference between the number of significant positive and negative correlations with DSN1 expression. Column “TMB”, tumor mutation burden. “+”/“-” means a positive/negative correlation between DSN1 expression and TMB (p < 0.05). Column “PD1”, “+”/“-” represents better/poorer overall survival for the high DSN1 expression group in the anti-PD-1/PD-L1 therapy cohort. ns, not significant; empty cell, data not available (NA).

Cancer	mRNA	Pr.	SC	TP53 Mut.	KM OS	Met.	MSI	Th2	ES	Mac.	IF	ISTF	ISUF	ICP	CK	CKR	TMB	PD1
ACC	ns			ns	+	ns	ns	+	ns	-	-8	13	2	0	4	3	+	
BLCA	+		T	+	ns	-	+	+	ns	+	-6	18	15	9	23	4	+	-
BRCA	++	+	MT	+	++	-	+	+	-	ns	-1	10	10	8	0	2	+	
CESC	+		M	ns	-	-	+	ns	-	-	-14	0	-2	-5	-12	1	ns	
CHOL	++		MT	ns	ns	ns	ns	ns	ns	ns	-2	0	0	0	0	0	ns	
COAD	++	+	T	+	+	-	ns	ns	-	-	-11	-5	-3	-1	2	-1	-	
DLBC	+			ns	ns	-	ns	+	ns	ns	2	8	3	0	-1	1	ns	
ESCA	+		MT	ns	ns	-	+	+	-	-	-8	3	-1	-2	2	-3	ns	
GBM	+	+	M	+	ns	-	+	+	-	-	-10	1	3	0	-7	-1	ns	ns
HNSC	++	+	MT	ns	+	-	+	+	-	ns	-7	21	9	3	7	3	ns	
KICH	+			ns	+	ns	+	+	ns	ns	-1	11	3	1	2	1	ns	
KIRC	++	+	T	ns	-	ns	ns	ns	ns	ns	0	34	18	7	20	13	ns	ns
KIRP	+			ns	+	-	+	ns	-	-	-15	10	4	0	-5	2	ns	
LGG	+			ns	+	-	+	+	ns	-	-2	26	18	9	5	13	+	
LIHC	++	+	MT	+	+	-	+	+	-	ns	-4	32	16	8	15	12	ns	
LUAD	++	+	T	+	+	-	+	+	-	ns	-14	2	7	4	6	-6	+	
LUSC	+	+	T	ns	-	-	+	ns	-	-	-22	-22	-9	-5	-25	-16	+	
MESO				ns	+	ns	ns	+	ns	ns	-4	11	3	1	-1	0	ns	
OV	++	+	MT	ns	+	ns	+	+	-	-	-12	26	18	8	9	9	ns	
PAAD	++	ns	M	ns	++	ns	+	+	ns	-	-4	13	8	4	1	1	+	
PCPG	ns			ns	ns	ns	ns	ns	ns	-	-2	12	5	1	4	3	ns	
PRAD	+		M	ns	+	-	+	ns	ns	ns	5	41	21	9	23	15	ns	
READ	+			+	-	-	+	ns	ns	-	-2	5	1	0	8	1	ns	
SARC	+			+	+	-	+	+	-	-	-19	-3	1	2	-5	0	+	
SKCM	+		T	ns	ns	-	+	+	-	ns	-11	10	8	3	3	1	ns	+
STAD	++		ns	+	--	-	+	ns	-	-	-19	-7	-1	-1	0	-10	+	
TGCT	-			ns	ns	-	ns	+	ns	ns	10	14	10	9	-14	5	ns	
THCA	ns		MT	ns	ns	ns	ns	ns	-	-	-16	5	-1	-4	-7	-1	ns	
THYM	+			ns	-	-	ns	+	-	-	-3	-6	0	-2	-16	-3	-	
UCEC	++	ns	MT	+	ns	ns	ns	+	-	ns	-12	7	2	-2	-2	1	ns	
UCS	+			ns	ns	ns	ns	ns	ns	-	-1	0	2	0	-1	-1	ns	
UVM			ns	ns	+	ns	+	+	+		11	37	21	10	25	14	ns	

## Abbreviations

ACC: Adrenocortical Carcinoma
BLCA: Bladder Urothelial Carcinoma
BRCA: Breast Invasive Carcinoma
CESC: Cervical Squamous Cell Carcinoma and Endocervical Adenocarcinoma
CHOL: Cholangiocarcinoma
COAD: Colon Adenocarcinoma
CPTAC: Clinical Proteomic Tumor Analysis Consortium
DLBC: Lymphoid Neoplasm Diffuse Large B-cell Lymphoma
DSN1: Dosage Suppressor of NNF1
ESCA: Esophageal Carcinoma
GBM: Glioblastoma Multiforme
GEO: Gene Expression Omnibus
GSEA: Gene Set Enrichment Analysis
GTEx: Genotype-Tissue Expression Project
HNSC: Head and Neck Squamous Cell Carcinoma
KICH: Kidney Chromophobe
KIRC: Kidney Renal Clear Cell Carcinoma
KIRP: Kidney Renal Papillary Cell Carcinoma
LAML: Acute Myeloid Leukemia
LGG: Lower Grade Glioma
LIHC: Liver Hepatocellular Carcinoma
LUAD: Lung Adenocarcinoma
LUSC: Lung Squamous Cell Carcinoma
MESO: Mesothelioma
MSI: Microsatellite Instability
OV: Ovarian Serous Cystadenocarcinoma
PAAD: Pancreatic Adenocarcinoma
PCPG: Pheochromocytoma and Paraganglioma
PRAD: Prostate Adenocarcinoma
READ: Rectum Adenocarcinoma
SARC: Sarcoma
SKCM: Skin Cutaneous Melanoma
STAD: Stomach Adenocarcinoma
TCGA: Cancer Genome Atlas
TGCT: Testicular Germ Cell Tumors
THCA: Thyroid Carcinoma
THYM: Thymoma
TISCH: Tumor Immune Single-cell Hub
TMB: Tumor Mutational Burden
UCEC: Uterine Corpus Endometrial Carcinoma
UCS: Uterine Carcinosarcoma
UVM: Uveal Melanoma
