# Supplementary material for: A Comprehensive Analysis of the Role of DSN1 in Pan-Cancer Prognosis and Immunotherapy
Source: J Cancer. 2025 Apr 21;16(8):2449–65. doi: 10.7150/jca.111585 (PMC12170500; doi:10.7150/jca.111585)
Supplement: Supplementary file 1 — Supplementary figures. [file jcav16p2449s1.pdf]

Supplement Fig. S1

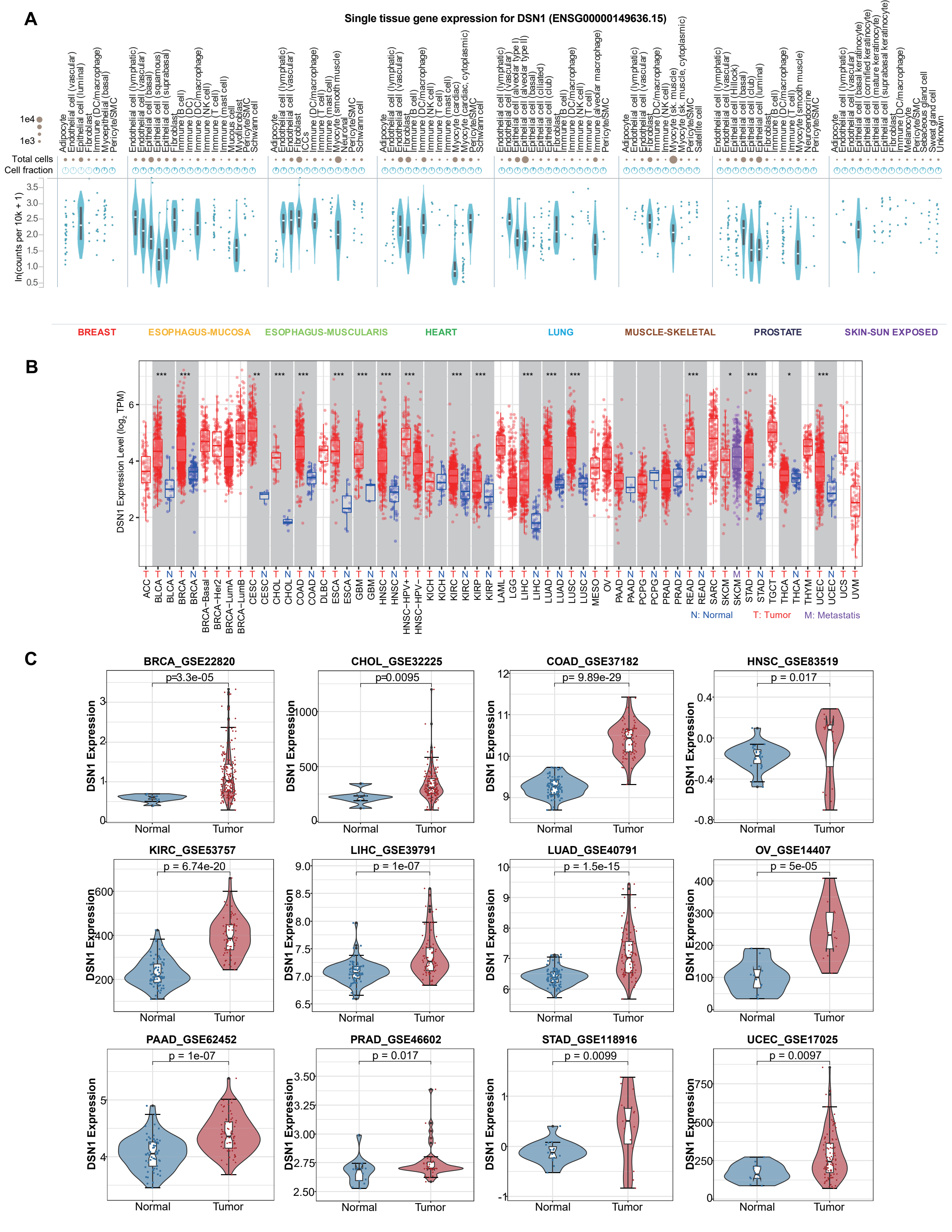

Supplementary Fig. S1:

Differential DSN1 expression across normal and tumor tissues.

(A) Violin plot illustrating single-cell expression levels of DSN1 in human normal breast, esophagus, heart, lung, muscle, prostate, and skin tissues.

(B) Box plot depicting DSN1 mRNA expression levels in normal and tumor tissues, based on data from the TCGA database.

(C) The violin plot shows the differences in DSN1 gene mRNA expression levels between different types of tumor tissues and normal tissues, based on the GEO database.

Supplement Fig. S2

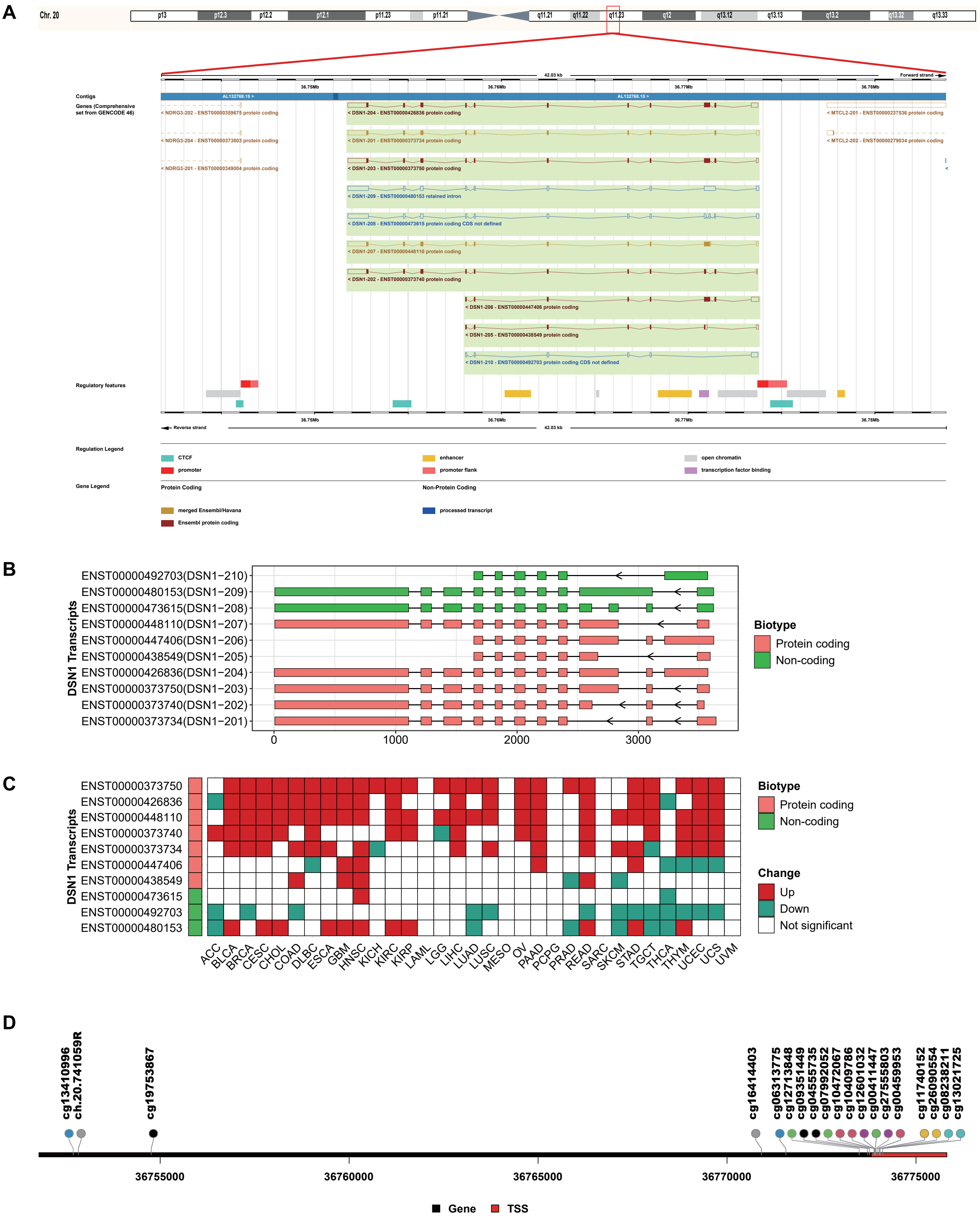

Supplementary Fig. S2:

Analysis of DSN1 transcript structure, expression differences, and methylation in tumor and normal tissues.

(A-B) Locations and transcript structure of 10 different DSN1 gene transcripts on Chromosome 20.

(C) Differential expression of DSN1 transcripts across 33 types of tumor and normal tissues. Red squares ($\log_2 FC > 0.5$) indicate upregulation in tumor tissues, while green squares ($\log_2 FC < -0.5$) indicate downregulation.

(D) Distribution map of methylation sites in the DSN1 gene region. Different colored dots represent various CpG sites, with the red box highlighting the transcription start site (TSS) region of the gene.

Supplement Fig. S3

A

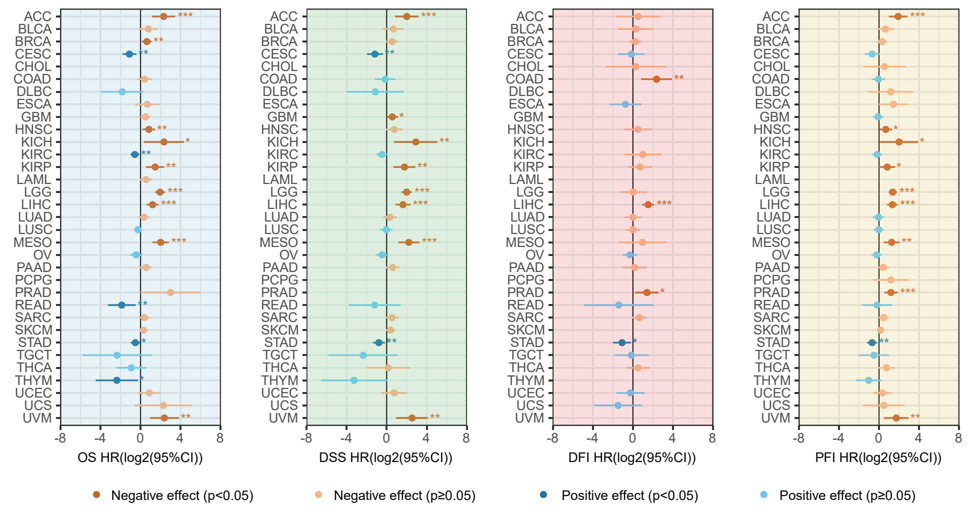

B

GEO-KM-OS

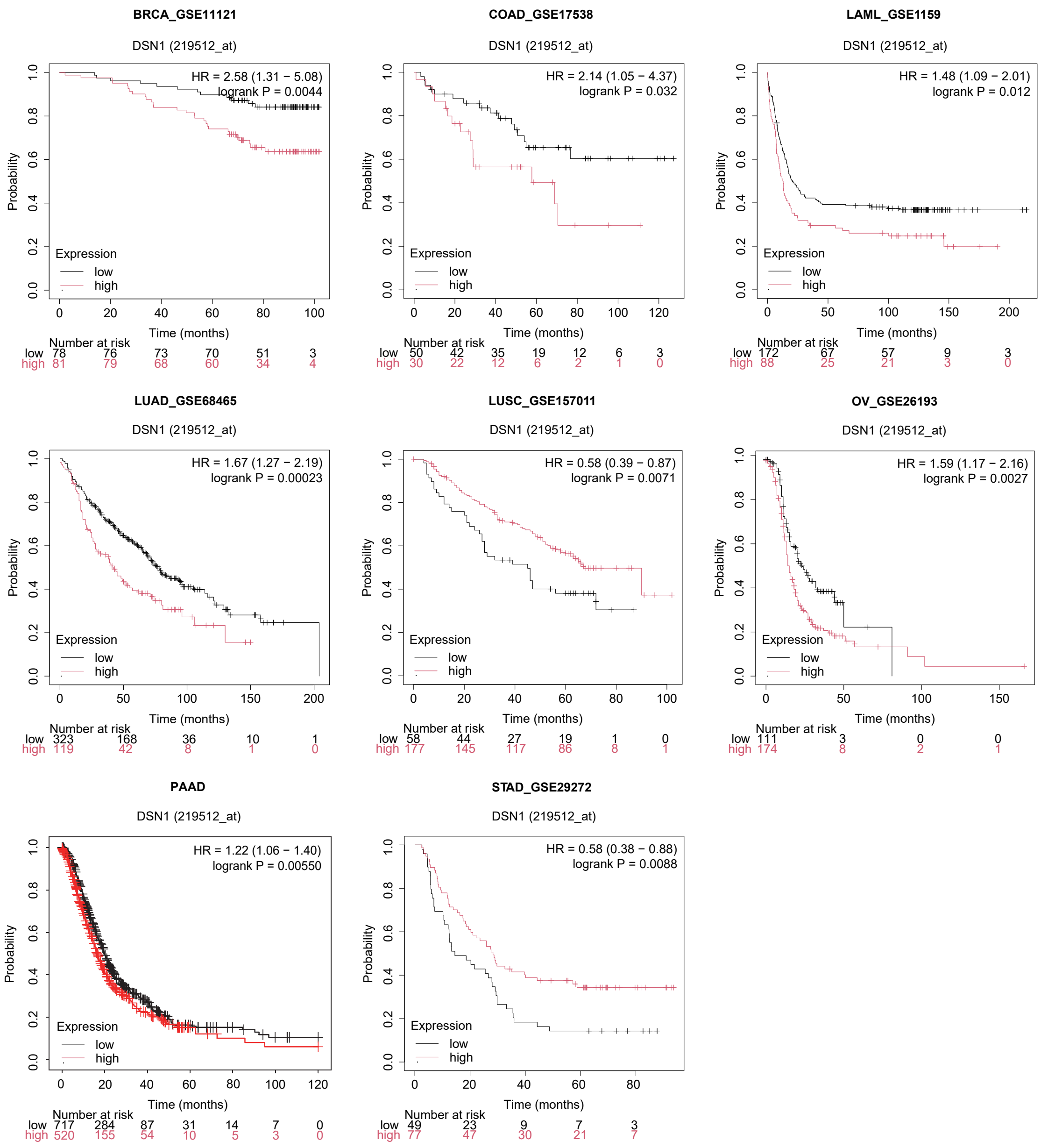

Supplementary Fig. S3:

Analysis of the association between DSN1 expression and survival prognosis in pan-cancer.

(A) The forest plot shows the results of univariate Cox regression analysis of DSN1 expression with OS, DSS, DFI, and PFI across 33 TCGA tumor types. The symbols *, **, and *** represent $P < 0.05$, $P < 0.01$, and $P < 0.001$, respectively.

(B) Survival curves comparing the prognosis of high and low DSN1 expression groups in BRCA, COAD, LAML, LUAD, LUSC, OV, PAAD, and STAD from the GEO database. Data were analyzed using the KM plotter online tool.

Supplement Fig. S4

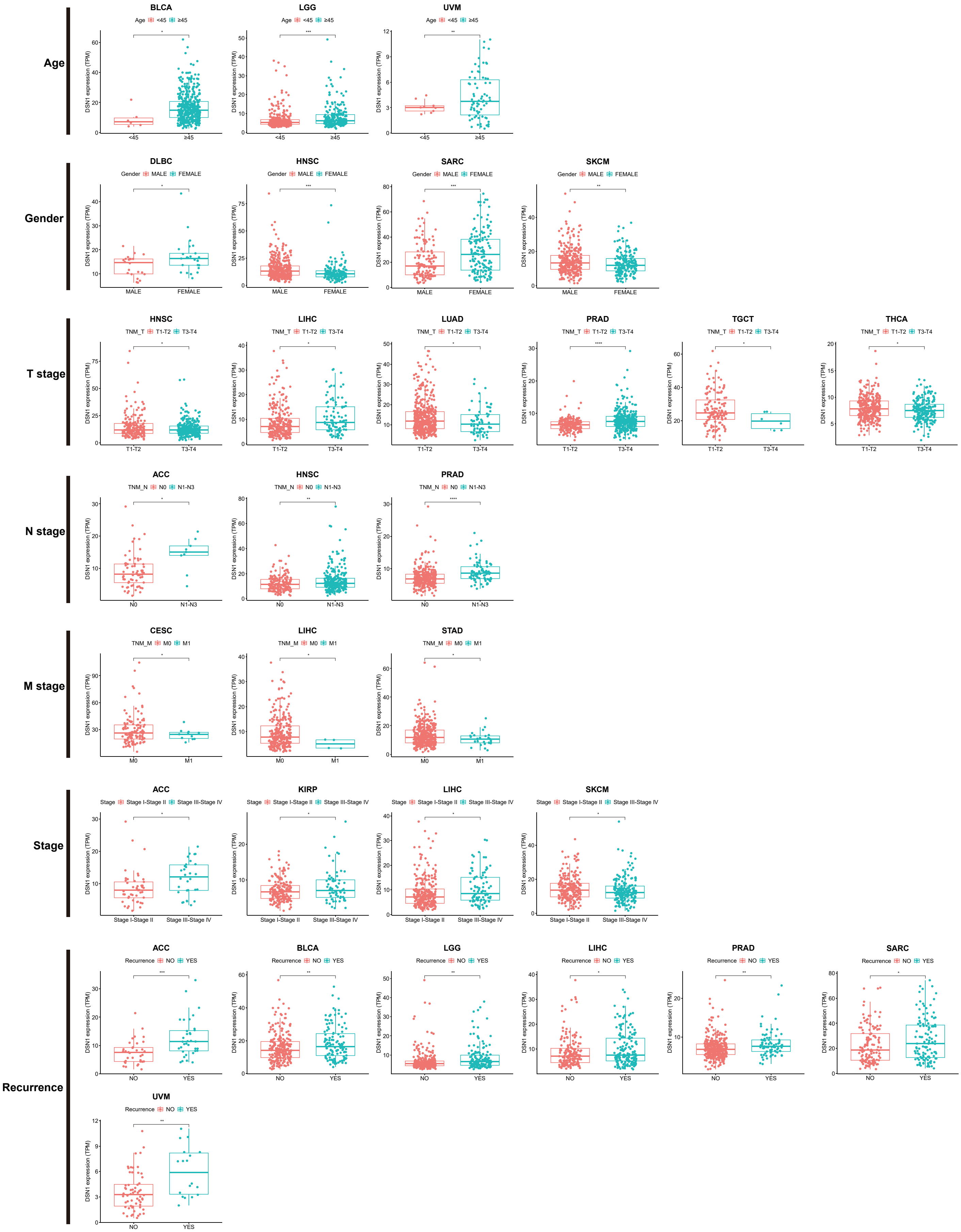

Supplementary Fig. S4:

Correlation between DSN1 expression and clinical characteristics of patients across pan-cancer.

Box plots show the correlation between DSN1 expression levels and seven clinical features, including age, gender, T stage, N stage, M stage, tumor stage, and recurrence, across various cancer types. The symbols *, **, ***, and **** represent $P < 0.05$, $P < 0.01$, $P < 0.001$, and $P < 0.0001$, respectively.

Supplement Fig. S5

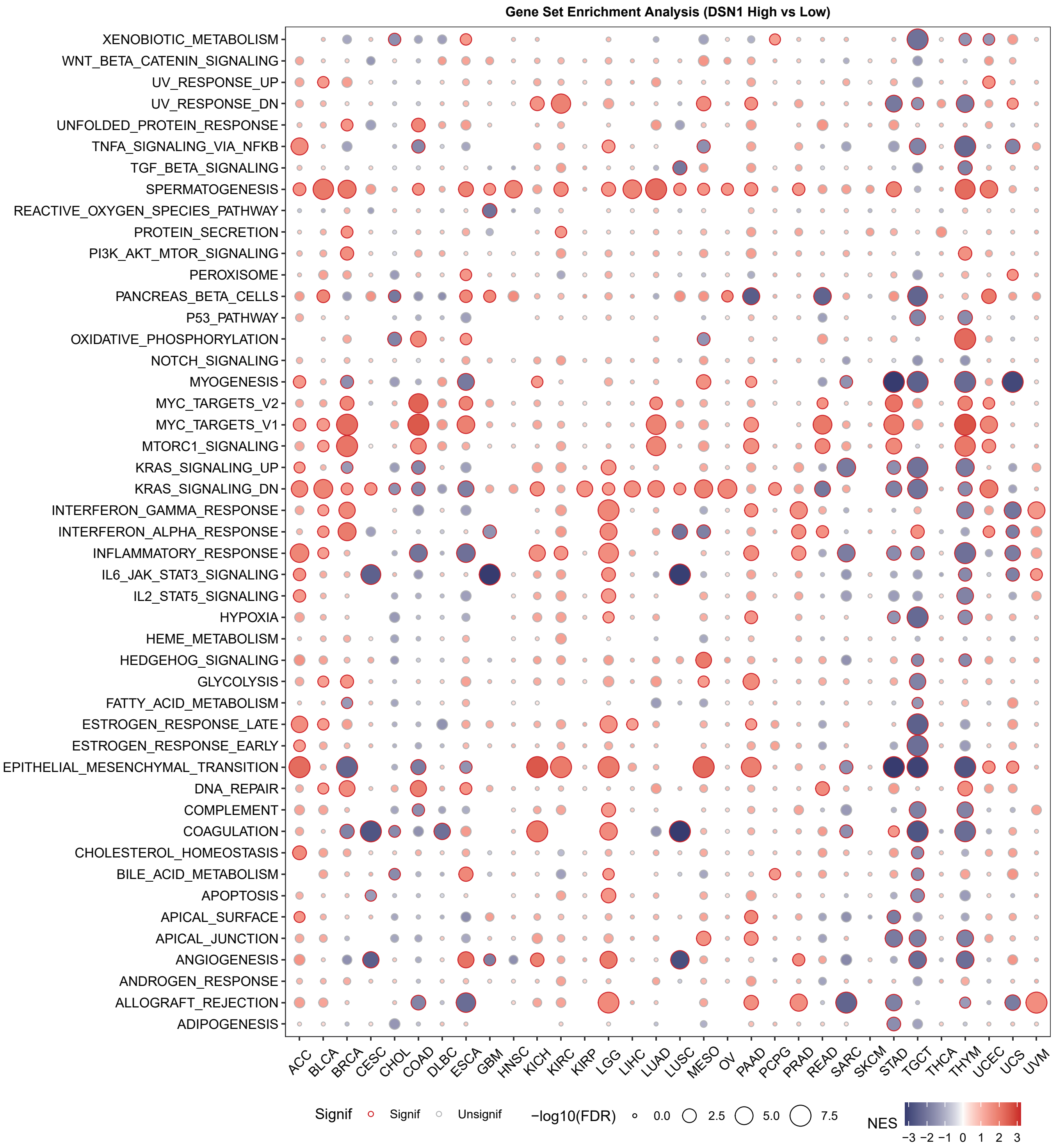

Supplementary Fig. S5:

Potential functional analysis of DSN1 in human cancers using GSEA.

The bubble plot shows the GSEA results between high and low DSN1 expression tumor patients using hallmark gene sets, with high and low groups defined by the top and bottom 30% of DSN1 expression levels. The size of each circle represents the magnitude of the P-value, while the color gradient from red to white to blue indicates the size of the normalized enrichment scores (NES).

Fig. S6

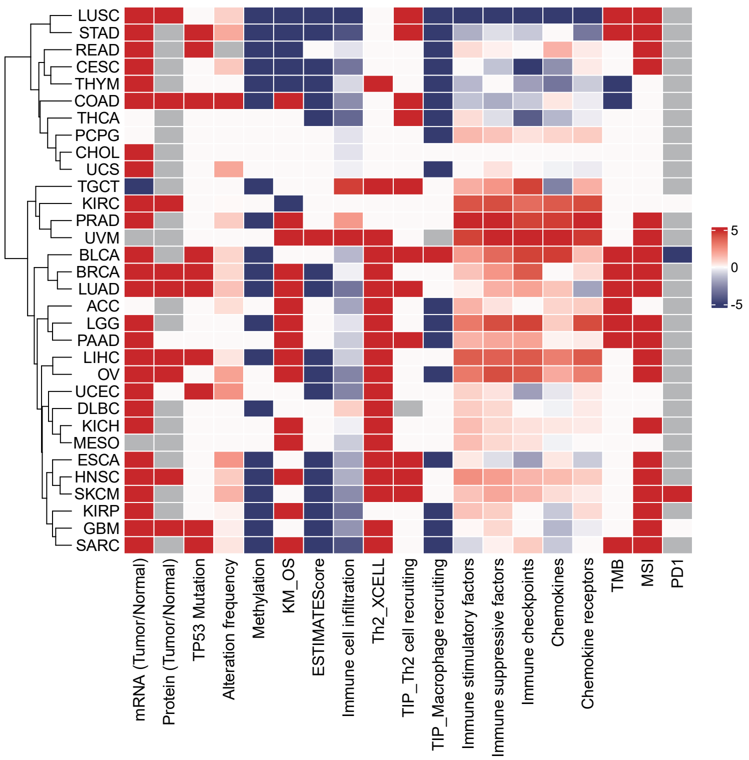

Supplementary Fig. S6:

Clustering heatmap of DSN1 biological features across different cancer types.

Based on the results from Table 1 and our study, the data for the heatmap were quantified as follows: “+” or “++”(red) for upregulation/positive correlation (value = 5), “-” or “--” (blue) for downregulation/negative correlation (value = -5), and “n.s.” (white) for no significant correlation (value = 0). Additionally, the data for columns such as “Alteration frequency”, “Immune cell infiltration”, “immune stimulatory factors”, “immune suppressive factors”, “immune checkpoints”, “chemokines”, and “chemokine receptors” were standardized and scaled to a range of [5 to -5].
